# Frequency-Based Professionalism Evaluation of (ing) and (t)-Deletion in England and Pennsylvania

**DOI:** 10.1177/00754242251343912

**Published:** 2025-06-30

**Authors:** Erik Schleef, Bradley Mackay, Jana Pflaeging

**Affiliations:** 1 University of Salzburg, Salzburg, Austria; 2 University of Michigan, Ann Arbor, MI, USA

**Keywords:** sociolinguistic monitoring, (ing), (T)-deletion, England, Pennsylvania

## Abstract

This study investigates the social evaluation of linguistic variation and the cognitive monitoring processes involved. Recognizing the need for cross-regional research that keeps experimental factors constant, we focus on (ing) and (t)-deletion. We investigate frequency evaluation as managed in England, UK (*N* = 200), and in Pennsylvania, US (*N* = 150). Results for (ing) indicate no significant effect of [ɪn]-frequency in England, while in Pennsylvania the frequency of [ɪn] significantly affects ratings regarding perceived professionalism. We also found evidence for listeners’ awareness of the attitude target (*N* = 15) to affect their ratings regarding perceived professionalism. Variation in (t)-deletion did not prompt any significant differences.

## 1. Introduction

Research in the sociolinguistic tradition is based on the assumption that variation is a normal feature of any language. Most research also makes assumptions about the cognitive abilities of speakers and hearers. For the majority of sociolinguistic research, this socio-cognitive theorizing has occurred mostly implicitly, for example when discussing how aware speakers are about certain types of variation and their respective ability to style-shift (Labov 1972). However, more recent research has tested some of the cognitive mechanisms involved in processing variable speech (e.g., Campbell-Kibler 2011; Levon & Buchstaller 2015; Vaughn & Kendall 2019; Vaughn 2022a, 2022b; García, Walker & Beaton 2023). One strand of research in this tradition revolves around the notion of ‘sociolinguistic monitoring’ as introduced by Labov, Ash, Baranowski, Nagy, and Ravindranath (2006) and Labov et al. (2011). In this paradigm, authors hypothesize that many sociolinguistic assumptions about language variation and social meanings can be explained if we assume that a monitoring process takes place during speech production and perception. In particular, researchers working in this paradigm have investigated how and why speakers and listeners track the speech signal for socially meaningful cues of variable features and monitor the frequency of these forms.

Much of this work has been perception- rather than production-oriented, in line with the original experiments carried out by Labov et al. (2011). In order to find out whether listeners are able to evaluate the fine-grained variation patterns that production studies have documented for (ing), they conducted a series of matched-guise experiments using a test passage consisting of short news items (164 words) with ten evenly-distributed progressive {*-ing*}-suffixes. Recordings were made with a female speaker of Standard American English, who articulated the focus words with two typical (ing)-variants: [ɪŋ] with a velar nasal and [ɪn] with an apical nasal (Labov et al. 2011:436-437). Seven guises were created by replacing none (0 percent), some (10 percent, 20 percent, 30 percent, 50 percent, 70 percent), or all of the [ɪŋ]-realizations (100 percent) with [ɪn] (Labov et al. 2011:436-437). Participants were told they would listen to seven versions of a trial newscast by a journalism student applying for a job with a local radio station. They were asked to rate each recording on a professionalism-scale, ranging from ‘perfectly professional (1)’ to ‘try some other line of work (7).’ Labov et al. (2011) found that guises with a larger number of [ɪŋ]-variants were judged to be more professional-sounding; recordings with more [ɪn]-variants were associated with lower professionalism-ratings.

Labov et al. (2011:434) remain “neutral” to the question of whether the tracking of sociolinguistic variation is carried out by “a separate processing and storage module” or is, for instance, done “‘on the fly’ at any time by an inspection of remembered tokens.” Instead, they elaborate on the characteristics of sociolinguistic monitoring conceptualized as listeners’ sensitivity to frequency variation in the production of linguistic variants and its influence on social evaluation. In describing these characteristics, Labov et al. (2011) focused on three issues: the time period across which frequency assessment accumulates, the number of tokens it is sensitive to and the distribution of the response. Regarding these three points, they found the time window to be at least fifty-seven seconds long. Listeners are sensitive to frequency differences of even one [ɪn]-token, and many listeners’ responses follow a logarithmic rather than a linear pattern, that is, evaluation is attenuated as more tokens of a form occur—each individual token has a reduced effect compared to the previous one. Wagner and Hesson (2014:656), who also focused on variation in (ing), implemented a similar research design and used the same audio-files as Labov et al. (2011), found a similar response pattern.

Several other replication studies on (ing) and other variables (e.g., (th)-fronting, Levon & Fox 2014; Levon & Buchstaller 2015; the Northern-Subject Rule, Levon & Buchstaller 2015) did not find the same logarithmic pattern. Instead, Levon and Fox (2014) as well as Levon and Buchstaller (2015) found evidence of a *linear* relationship between the number of non-standard tokens and listener evaluation (Levon & Fox 2014; Levon & Buchstaller 2015). This means that each token impacts the evaluation equally and that even at higher non-standard rates social judgments are made. Other studies even attested a flat, non-significant response rate (Levon & Fox 2014; Stecker 2020; García, Walker & Beaton 2023), which indicates that listener evaluation was unaffected by quantitative differences in non-standard variants.

Levon and Fox’s (2014) replication of Labov et al.’s study in London is particularly relevant to our study because of its interest in regional differences in the evaluation of (ing)-based frequency variation between the US and the UK. Despite using a slightly longer, adapted test passage, they followed Labov et al.’s original study design and also focused on professionalism ratings. As listener evaluations were entirely unaffected by how many [ɪn]-tokens a guise included (Levon & Fox 2014:197-198), the authors conclude that “listener attitudes to (ing) on the professionalism-dimension are weaker in Britain than in the United States” (Levon & Fox 2014:199) and that listener attitudes toward (ing) are more automatically accessible in the US than they are in Britain (Levon & Fox 2014:199).

Any conclusions that relate to cross-regional patterns and attitude strength, however, remain speculative to some extent. While results from similar experiments in different locales may suggest general tendencies, Labov et al.’s (2011) original study and replications in the US context (Wagner & Hesson 2014) on the one hand and the British experiments on the other (e.g., Levon & Fox 2014) are not directly comparable. The speakers, the test passages, and the placement of (ing) all differed between these studies. What is needed for a balanced comparison of listener evaluation of quantitative variation in (ing) across different locales is a cross-regional study that keeps the speaker, test passage and recordings, as well as the overall survey design constant.

In order to address this research gap, we conducted a cross-regional study with the same adapted news-report and speaker throughout to compare listener evaluations of (ing)-based quantitative variation in three different locales: Pennsylvania (in the US) and the North as well as the South of England. In addition to (ing), we included (t)-deletion (Wells 1982; Labov 2001; Hazen 2006) as a further stable variable that is also found to be involved in style-shifting. It also creates a backdrop against which the evaluation of (ing) can be compared, as we suspect that participants are less likely to become aware of (t)-deletion during experimentation. Variation in (ing), in turn, is much more likely to raise participants’ awareness since it continues to receive overt public comment (Labov 1972). Vaughn (2022b:517), for instance, who also worked in the sociolinguistic-monitoring paradigm, reports that most of her participants guessed that her experiments focused on variation in (ing). Of course, repeated exposure to highly similar guises, as implemented in this paradigm, may increase the likelihood of listeners becoming aware of the attitude target. In Austen and Campbell-Kibler’s (2022) real-time study, which *did not* involve listening to multiple similar versions of a test passage, participants did not become aware of (ing) as the attitude target.

Experimenting with stereotypical features such as (ing) presents researchers with a methodological problem as study participants’ awareness of the experimental target may influence evaluation. Much attitude research actually recommends removing participants who have realized what the study is about (e.g., Kircher 2016). However, the extent to which experimental participants become *aware* of the attitude target (be it a stereotyped feature or not) and how this varies from region to region is not known. This is why we will explore participants’ awareness of the attitude target, as indicated by listeners’ ability to name the attitude target when asked to do so (see section 3.3) and analyze the effects their (un)awareness has on the evaluation of variants.

By doing so, this study contributes to a better understanding of the listener characteristics that may impact evaluation as it investigates the impact of listeners’ regional background and their potential awareness of the feature under scrutiny. In particular, we explore

(1) whether there is a significant effect of quantitative variation in (ing) and (t)-deletion on listener evaluation in several regions (i.e., Pennsylvania, the North of England, and the South of England),
(2) what the nature of the response pattern is (i.e., is it logarithmic or linear), and
(3) in how far listeners’ (un)awareness of the attitude target affects their evaluation.

Thus, our motivation for this study is, first and foremost, of a comparative nature. We conduct a cross-regional replication study in the tradition of sociolinguistic-monitoring experiments to provide alternative explanations for the contradictory findings made in previous research.

Although our focus is a cross-regional comparison that relates to Labov et al.’s (2011) original study, our own view about *sociolinguistic monitoring* differs somewhat from that of Labov et al. (2011:434) whose conception relates to both speech production and perception. During the last decade, research has provided evidence of the disjunct of production and perception (e.g., Levon 2014; Schleef 2023), in particular when approaching language variation from a third-wave perspective. While research on style production has argued that features cluster into styles to create social meanings (Eckert 2012), it is by no means clear that listeners can actually perceive all features equally and recognize the intended meanings. In addition, Campbell-Kibler (2016; also Austen & Campbell-Kibler 2022) has been critical of Labov et al.’s (2011) proposal for being unrealistically broad in covering both self-regulation and other-perception. Instead, sociolinguistic monitoring of production and perception may be two distinct (although possibly interrelated) processes that are impacted by various systems to differing degrees. This is why our own conception of sociolinguistic monitoring in this paper is limited to perception, without making assumptions about production. Particularly, we agree with Campbell-Kibler (2016; also Austen & Campbell-Kibler 2022), who argues that there is no need for specialized cognitive modules to explain sociolinguistic behavior and that cognitive structures proposed elsewhere are capable of accounting for it (see Austen & Campbell-Kibler 2022:128).

## 2. The Variables

### 2.1. (ing)

Variation in (ing) generally refers to the varying spoken realization of the progressive {*-ing*}-suffix as either [ɪŋ] or [ɪn], as in *making*. These two main variants occur in almost all varieties of English. Both in the UK and the US context, variation in (ing) is constrained by a combination of internal and external factors (e.g., Trudgill 1974; Labov 2001; Hazen 2006; Schleef, Meyerhoff & Clark 2011). Previous research shows that there are phonological environments that favor the velar variant [ɪŋ] (Houston 1985; also Hazen 2006:583; Schleef, Meyerhoff & Clark 2011:235). Based on her British data, Houston (1985:19-20) concludes, for instance, that [ɪŋ] is more likely to be produced when the following segment begins on a velar sound. With regard to previous segments, a preceding velar stop or apical is the strongest predictor of an apical realization of (ing). Hazen (2006:583, in ref. to Houston 1985:22) also collates arguments for stress placement as a predictor of [ɪŋ] or [ɪn], respectively. In multi-syllabic words, unstressed syllables tend to be realized as [ɪn], whereas a secondary stress tends to correlate with a velar realization of (ing). Moreover, due to the historical development of (ing), there is also grammatical conditioning with regard to the morphological class of the {-*ing*}-form. Verbs have been shown to favor the [ɪn]-variant, whereas [ɪŋ] usually occurs in nouns (Houston 1985; Labov 2001:86-87; Vaughn 2022b:513; Tagliamonte 2004).

The sociolinguistic constraints operating on (ing) have been relatively well described (e.g., Campbell-Kibler 2007, 2009, 2011; Labov et al. 2011; Schleef & Flynn 2015; Schleef, Flynn & Barras 2017; Bailey 2019). Generally, the apical variant [ɪn] is more frequent in informal styles, male and working-class speech. However, there are more specific patterns that result from interactions with region and the baseline frequency of [ɪn] in a particular area (e.g., Labov 2001:90). In the UK, especially, we find regional differences in the use of (ing). Based on VARBRUL probabilities, Houston (1985:108) suggests there are two dialect groups: the southern/internal group, favoring [ɪŋ], and the northern/peripheral group, favoring [ɪn]. Houston (1985:103), focusing on working-class speech, cites [ɪŋ]-values of 20-42 percent for London, 21 percent for Manchester, and 18 percent for Edinburgh. This resonates with findings by Tagliamonte (2004), who attests little symbolic social value attached to (ing) in the Northern English city of York. Social class is the only social factor that reaches statistical significance, and its effect size is not particularly strong. For a number of socio-historical reasons, the use of (ing) is more socially conditioned in southern varieties of British English (Schleef & Flynn 2015:52; Schleef, Flynn & Barras 2017:32-33, in ref. to Tagliamonte 2004:401), where the [ɪŋ]-variant is the prestigious form and tends to be used by middle- and upper-class speakers (Levon & Fox 2014:195). Similar cases of a stronger sociolinguistic conditioning have been reported for the US context (Tagliamonte 2004:401).

Perception-oriented research on the evaluation and social meanings of (ing) has been equally prolific. Campbell-Kibler (2007:32-33, 2011:423) found that US speakers who produce [ɪŋ] are normally rated as significantly more intelligent, educated, articulate, and less likely to be a student. The use of [ɪn], in turn, was associated with informality and speakers were rated as less educated, more likely to be from the country, and less gay-sounding. In the UK context, such patterns have also been shown to interact with social class as well as region. Middle-class listeners, independent of region, agree on the formality of [ɪŋ] (Schleef, Flynn & Barras 2017:46). However, working-class participants in different locales in the UK may hold different attitudes for some social-status scales. Research within the sociolinguistic-monitoring paradigm mentioned above has also contended that the evaluation of [ɪn], regarding professionalism, is less negative in London (Levon & Fox 2014) than in the US context (Labov et al. 2011).

### 2.2. (t)-Deletion

(t)-deletion refers to the presence or elision of the morpheme-final plosive /t/, which, for example in a word like *best*, results in two main pronunciation variants, namely *best* [bɛst] and *bes’* [bɛs]. As with variation in (ing), (t)-deletion occurs in almost all varieties of English (Tagliamonte & Temple 2005:282). Studies investigating the UK context show that [t] is regularly deleted in locales such as York (24 percent, Tagliamonte & Temple 2005:287-288) and Manchester (37 percent, Baranowski & Turton 2020:15). Likewise, Pavlík (2017) attests the deletion of /t/ in 26 percent of words ending in *-n’t* or *-nt* in the Standard British English of BBC newscasters. In the US, usage rates are generally similar, at 33 percent (Guy 1991). The variability of (t)-deletion, however, is conditioned by a diverse set of internal and external factors. Not least for this reason, (t)-deletion is among the most extensively studied variables in sociolinguistic research (Pavlík 2017:195; Baranowski & Turton 2020:1), typically in combination with a focus on (d)-deletion as a closely related phenomenon.

Phonological environment has been found to be one of the strongest predictors of (t)-deletion. The variant deleted-[t] typically occurs when preceded by sibilants (then stops, nasals, other fricatives, and liquids, see Labov 1989). Further phonological environments that favor deleted-[t] are following consonants or non-sonorous segments; following vowels and sonorants inhibit deletion-processes (Labov 1989; Guy 1991; Tagliamonte & Temple 2005; Smith, Durham & Fortune 2009; Hazen 2011).

The variability in (t)-deletion may also be a function of higher-level linguistic constraints such as the morphological structure and class of a word (Guy 1980, 1991; Tagliamonte & Temple 2005; Baranowski & Turton 2020), with underived, mono-morphemic words favoring deleted-[t] (summarized in Tagliamonte & Temple 2005:284, 290) and inflected verb forms disfavoring it (Guy 1991; also Pavlík 2017:197). While numerous studies attest these patterns for the US context (Baranowski & Turton 2020:3, 4; see e.g., Guy 1991), results for the UK context paint a more complex picture. Tagliamonte and Temple (2005:290), for instance, did not find a significant effect of word class on (t)-deletion in York. In a large-scale replication study in Manchester, however, Baranowski and Turton (2020) did find morphological class to predict (t)-deletion in ways similar to the US context.

Among the *sociolinguistic* factors that have been found to condition (t)-deletion are ethnicity (Fasold 1972; Hazen 2011), age (Hazen 2011), social class (Fasold 1972; Hazen 2011), and gender (Tagliamonte & Temple 2005), though the impact of these factors varies. In data from York, for instance, male participants were found to delete slightly more frequently (Tagliamonte & Temple 2005), while there was no gender difference in Manchester (Baranowski & Turton 2020:15-16). Social class and age were also not significant in Manchester, but stylistic differences were found with less deletion associated with more formal styles.

As this brief survey suggests, despite the comparably rich description of the variability of (t)-deletion in English overall, much less is known about (t)-deletion in British English (Tagliamonte & Temple 2005; Pavlík 2017; Baranowski & Turton 2020) compared with American English (e.g., Wolfram 1969; Fasold 1972; Guy 1980; Labov 1989; Bayley 1994; Santa Ana 1996; Coetzee & Kawahara 2013). This situation calls for further work on (t)-deletion in the UK context as well as comparative studies across regions. In addition, it is aspects of style and the social evaluations of (t)-deletion, which seem to demand much closer attention. Our study seeks to explore both avenues of research.

### 2.3. Hypotheses

In this study, we first explore the effect of quantitative variation of token frequency on listener evaluation across several locales. As both (ing) and (t)-deletion are reported to be involved in style-shifting, we expect that there is a significant effect of frequency differences in all regions. However, given the indication of weaker attitudes in Britain (see Levon & Fox 2014), the effect size will likely be smaller for (ing) in England. Our review of variables also suggests that the difference in social stratification and general commentary is less pronounced for deleted-[t] than for use of [ɪn] in both Britain and the US. As a result, we would expect a smaller effect of variant frequency for (t)-deletion than for (ing).

Secondly, we investigate the nature of the response pattern in the three different locales. Given previous findings for the UK and the US context, we also expect differences in the nature of the response pattern for (ing), but not necessarily for (t)-deletion. For (ing), a linear pattern seems to be most likely for responses from UK listeners and a more logarithmic distribution for responses in the US as it has been argued that attitudes toward variation in (ing) are weaker in Britain than they are in the US.

We thirdly investigate in how far listeners’ (un)awareness of the attitude target affects their evaluation. We hypothesize that, in the case of (ing) especially, individuals who have become aware of the attitude target (i.e., what the experiments are testing) will evaluate quantitative variation differently from those participants who have not. In order to test these hypotheses, we conducted experiments on the perception of (ing) and (t)-deletion in England and Pennsylvania.

## 3. Methods

### 3.1. Test Passages

The design of our research and test materials closely follows Labov et al.’s (2011) original study. We also chose the social context of a news broadcast and kept the token number at 10 progressive {*-ing*}-suffixes. At ca. 1.2 minutes, our test passage was slightly longer than the original passage. The text that was used (see Appendix, section “Test Passages”) was inspired by an actual on-site news report (aired as part of *BBC Breakfast* in 2018, see Fritz 2018), which focused on prefabricated housing, a relatively neutral topic. The original script, however, was changed considerably to ensure that [ɪn] and deleted-[t] occur in plausible and consistent linguistic environments.

Looking at the linguistic contexts more specifically, variation in (ing) was limited to unique bi- or tri-syllabic present-participle forms, for example, *testing*, *working*, *referring*. The decision to focus on verb forms rather than nouns was motivated by previous research that has found the realization of (ing) as [ɪn] to be more likely in present-participle forms than in nouns that end in {*-ing*} (Houston 1985; Labov 2001:86-87; Vaughn 2022b:513; Tagliamonte 2004). As some words in English can occur as a present-participle and also as a noun, for example, *being* (but cf. *putting*), all relevant token words were checked for frequency of occurrence in the spoken BNC (see Vaughn 2022b:513). Only those words that are more commonly used as verb-forms in spoken British English were considered when creating the test passage; word-forms that are predominantly used as nouns, for example, *building*, were avoided entirely. We selected non-velar consonants (i.e., /b/, /f/, /ð/, /θ/, /s/, /h/) as following phonological segments, which are contexts that favor [ɪn] (Houston 1985:19-20; also Hazen 2006; Schleef, Meyerhoff & Clark 2011:235) or show no significant effect (Tagliamonte 2004:399). In addition, we spaced out target tokens in relatively equal distances of at least twelve word-forms and avoided other words ending in {*-ing*}.

Variation in (t)-deletion was restricted to mono- or bisyllabic words, for example, *best*, *grant*, *strongest*, and limited to /t/ in word-final position. Given the phonological contexts that favor deleted-[t] in this position (Baranowski & Turton 2020:9-11; also Labov 1989; Guy 1991; Tagliamonte & Temple 2005; Smith, Durham & Fortune 2009; Hazen 2011), all target tokens have either /s/ or /n/ as a preceding sound. The onset of the following word was limited to certain obstruents (i.e., /k/, /p/, /f/). Moreover, the onset of the following word had to differ from the new coda of the target token (once [t] is deleted), that is, constructions such as *bes’ suggestions* were avoided. As with the (ing)-tokens, we excluded other word-forms ending in /t/ and ensured a minimum distance of thirteen word-forms between (t)-deletion tokens.

### 3.2. Speaker Profiles and Guise Construction

To create the main set of guises, test passages were recorded with a male L1 speaker of British English with a southern accent. We also recorded a male L1 speaker of Standard American English from Rhode Island, who grew up in New Jersey and lived in Pennsylvania for several years. This guise was used only with US listeners to explore the effect of accent on evaluation. Both speakers were in their mid-thirties. For the recordings, both speakers produced a first version of the text with consistent use of [ɪŋ] and then again realizing all (ing)-tokens as [ɪn]. The procedure was repeated for both (t)-variants, first with word-final [t] and then with deleted-[t]. All recordings were made in a sound-attenuating booth at the University of Salzburg. Guises were constructed through digital splicing in *Praat* (Boersma & Weenink 2022), which involves replacing a given pronunciation variant, for example, [ɪŋ], with an alternative variant, for example, [ɪn]. In case of the news-reporting experiments, guise construction involved replacing either none (0 percent) or one (10 percent), two (20 percent), three (30 percent), five (50 percent), seven (70 percent), or all (100 percent) standard variants with the non-standard ones.^1^ In all cases, splicing concerned the sounds in question; only in very few cases did we have to replace small parts of the immediate phonetic environment. All recordings were scaled to 70 db.

### 3.3. Procedure and Questionnaire

The data was collected October-December 2023 through paid web-based experiments using *Lime Survey* (LimeSurvey GmbH 2023). In each news-reporting experiment, participants evaluated the seven different versions of the (ing) or the (t)-deletion test passage. Following established practice (e.g., Labov et al. 2011:444), the 50-percent guise was always heard first as a point of reference; all other guises were randomized and set to auto-play from start to finish. Participants could only proceed to the next page once a recording had played through and was scored. Similar to the original study, scores were recorded using a 7-point rating scale ranging from *try some other line of work* (1) to *perfectly professional* (7).

This section was followed by items assessing respondents’ awareness of the attitude target. Participants were given the question prompt *If we told you this study was about a specific language feature, which one would you think it was?* and asked to respond to it by entering a short answer into a textbox. Responses were subsequently jointly coded by two researchers, who considered any instances of participants explicitly mentioning the attitude target as an indication of awareness (e.g., *I’d say the “ing” sound. Throughout these recordings the speaker has often said it as “in” instead*, or also *Including or dropping the ‘g’ sound at the end of a word such as reporting, thinking, etc.*). Whenever responses did not explicitly mention the feature under scrutiny (e.g., participants stating that the study was about *inflection and pacing*, or about the *construction industry*, or responded with *don’t know*), the respective participant was considered unaware of the attitude target. Unclear cases were discussed with a third researcher to support the decision-making process.

The surveys also included identification tasks, which tested participants’ ability to identify the variants in focus. Listeners were presented with short test sentences (e.g., *She was running in order to catch the bus.*) after being told that a given focus word (e.g., *running*) would be produced with either one variant or another (e.g., [ɪŋ] or [ɪn], see also Schleef, Roth, Mackay & Pflaeging forthcoming).^2^ Listeners heard the recording of the sentence, and they were asked to identify the variant in the recording. The questionnaires also contained questions relating to participants’ own use of (ing)- and (t)-deletion variants, along with three psychometric tests. The *Revised Self-Monitoring Scale* (SMS, see Cramer & Gruman 2002) assesses study participants’ self-monitoring habits (subscale “Ability to Modify Self-Presentation,” i.e., amsp) and their “Sensitivity to the Expressive Behavior of Others” (subscale sebo). We believe that these participant characteristics are related to processes of sociolinguistic monitoring. The *Broad Autism Phenotype Questionnaire* (BAPQ, see Hurley, Losh, Parlier, Reznick & Piven 2007) collects information on listeners’ cognitive style, e.g., their preference for patterns and repetitions. It contains subscales, such as pragmatic language, which assesses an individual’s ability to communicate effectively in social situations and to hold a fluid, reciprocal conversation (Hurley, Losh, Parlier, Reznick & Piven 2007:1681). The BAPQ has been implemented in other studies within the sociolinguistic monitoring paradigm as the skills it tests are assumed to be implicitly involved in forming social judgments on language variation (see Wagner & Hesson 2014:655, also Levon & Buchstaller 2015). The third psychometric test was the *Motivation to Control for Prejudiced Reactions* survey (MCPR, see Dunton & Fazio 1997), which also tests for listener characteristics that may equally be linked to social evaluation processes. Finally, we also collected participant demographics. The full survey with question prompts and answer options is reproduced in the Appendix (section “Questionnaire”). Further information on the psychometric tests implemented in this study can be found at Schleef, Mackay & Pflaeging 2025.

### 3.4. Participants

Experiments involved 350 participants across all surveys (see Table 1), who were recruited through *Prolific* (www.prolific.com). Participants from the North of England (*N* = 100) were either from the North East, North West, or Yorkshire and the Humber. Respondents from the South of England (*N* = 100) were from London, the South East, and the South West. US participants were from Pennsylvania (*N* = 150), to match the main region studied in Labov et al.’s (2011) original experiment.^3^ Using *Prolific*’s in-built screening function, we also filtered the pool of available participants further to ensure that all respondents were born and still live in these areas and that their first language was English. We also screened for respondents who, before turning eighteen, had spent most of their time in the respective locales. Participants in our experiments were 41.9 years of age on average (SD = 13.7), ranging from eighteen to eighty-three years, balanced for gender (based on *Prolific’s* categories), and with various employment statuses (e.g., not a student, full- or part-time employment, retired).

**Table 1.** Overview of Number of Participants per feature, speaker, and region

Feature	Speaker	Region	Participants (*N*)
ING	Speaker 1 (British English)	England (North)	50
ING	Speaker 1 (British English)	England (South)	50
ING	Speaker 1 (British English)	US (Pennsylvania)	50
ING	Speaker 2 (American English)	US (Pennsylvania)	50
TDEL	Speaker 1 (British English)	England (North)	50
TDEL	Speaker 1 (British English)	England (South)	50
TDEL	Speaker 1 (British English)	US (Pennsylvania)	25
TDEL	Speaker 2 (American English)	US (Pennsylvania)	25

### 3.5. Data Analysis

Professionalism ratings were submitted to linear mixed-effects regression models built using the *lmerTest* package (Kuznetsova, Brockhoff & Christensen 2017) run in *R* (R Core Team 2022). A maximal model containing all relevant dummy-coded categorical factors (including e.g., gender, age group, class) and continuous factors (e.g., mood) was manually stepped down, using R’s anova() function to compare models, with only those factors which improved the model fit being kept in the final model.^4^ The following overview is a full list of the factors considered when fitting the models (in alphabetical order); levels are given as they were included in the models.

- age group: younger (≤ 35) | middle (36-65) | older (≥66)
- audio: headphones | in-built computer speakers | external speakers
- awareness: yes | no
- broad autism phenotype questionnaire:
- 1 (=very rarely) | [. . .] | 6 (=very often): subscales: pragmatic language, rigid, aloof (Z-scored)
- class: lower working-class | lower middle-class | upper working-class |
- upper middle-class
- gender: man | woman
- guise (i.e., if participants heard 0 percent, 10 percent, 20 percent, etc. of tokens of [ɪn] and deleted-[t], respectively): 0 percent | 10 percent | 20 percent | 30 percent | 50 percent | 70 percent | 100 percent
- identification tasks: correct | wrong
- mood: −5 (=in a very bad mood) | [. . .] | +5 (=in a very good mood) (Z-scored)
- motivation to control for prejudiced reactions scale: 1 (=strongly disagree) | [. . .] | 7 (=strongly agree) (Z-scored)
- self-monitoring scale:
- 1 (=strongly disagree) | [. . .] | 5 (=strongly agree): subscales: amsp, sebo (Z-scored)
- usage: Yes, regularly. | Yes, sometimes. | No, never.

For the linear models, professionalism ratings were predicted as professionalism rating ~ guise. The logarithmic models were fit using *R*’s log() function: professionalism ratings were predicted as professionalism rating ~ log(guise).^5^ The final models included random intercepts for respondent. We also checked for interactions between guise and all other predictors. Model-fit checks were carried out through the check_model() function from the *performance* package (Lüdecke, Ben-Shachar, Patil, Waggoner & Makowski 2021) and the *report* package (Makowski, Ben-Shachar, Patil & Lüdecke 2021).

Results are based on SpeakerRegion-specific data sets, which reflect which speaker (British or American) was heard in which geographical region (North of England, South of England, Pennsylvania). We refer to them as BritNorth, BritSouth, BritPenn, and AmPenn, respectively. Following common practice in attitudes research (e.g., Kircher 2016), we initially excluded those participants from the data sets who had become aware of the attitude target. In a second step, we deliberately included these participants in the statistical analysis but tested for an interaction effect between guise and awareness to determine if participants’ awareness impacts their evaluation as the number of non-standard tokens increases.

## 4. Results

### 4.1. (ing)

Results for (ing) are based on four related experiments: using the same general research design throughout, we collected responses of fifty participants from the North of England, fifty respondents from the South of England, and another group of fifty participants from Pennsylvania, US. These experimental groups all heard and rated the British speaker (i.e. BritNorth, BritSouth, BritPenn). In addition, we collected responses from another fifty participants from Pennsylvania, who heard and evaluated the American speaker (i.e. AmPenn). Based on our assessment of participants’ awareness of the attitude target, we excluded fifteen participants altogether who had realized what the study was about (BritSouth: three listeners removed; BritNorth: six listeners; BritPenn: one; AmPenn: five).

**Figure 1.**
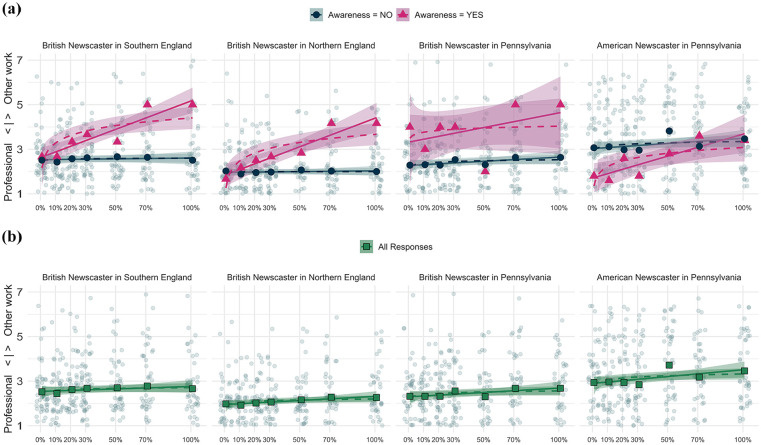
Professionalism rating by (ing) guise per SpeakerRegion. Upper Graphs (a): Pink Triangles = Mean Evaluations per Guise for Those Who Became Aware That (ing) was Being Targeted (*N* = 15). Blue Circles = Evaluations From Those Who Were Not Aware (*N* = 185). Linear Model Fits Are Shown With the Unbroken Lines and Logarithmic Fits With Dashed Lines. Lower Graphs (b): Green Squares = All Responses

Results indicate that listener evaluations in England were unaffected by guise, regardless of the locale in which guises were heard: both in the South of England (*Est.* = −0.0002, *p* = .86, see Table 2) and in the North of England (*Est.* = −0.001, *p* *=* .59, see Table 3), an increase in [ɪn]-tokens did not have an impact on perceived professionalism. The response patterns in both regions are flat (see Figure 1, upper graphs (a), dark blue lines with circles), which is further supported by non-significantly lower AIC values for the linear models for each locale (linear model for BritSouth: AIC = 832, *r*^2^ = 74.00; logarithmic model for BritSouth: AIC = 834, *r*^2^ = 74.00 and the linear model for BritNorth: AIC = 755.8, *r*^2^ = 45.00; logarithmic model for BritNorth: AIC = 756.1, *r*^2^ = 45.00).

**Table 2.** (ing). Results of the Linear Mixed-Effects Model Predicting professionalism rating by (ing) guise for the British Speaker in the South of England (*N* = 47)

Factors	Std. Error	Estimate	*df*	*t* Value	*p*-value
(Intercept)	0.24	2.45	47	10.42	<0.001***
guise	0.001	−0.0002	280	−0.18	0.86
age group = younger	0.33	−0.13	43	−0.38	0.70
age group = older	1.11	2.22	43	2.01	0.05
sebo	0.30	−0.44	50	−1.47	0.15
guise : sebo	0.002	−0.004	280	−2.17	0.03*

Asterisks in statistical testing indicate significance levels: *p < 0.05, **p < 0.01, ***p < 0.001.

**Table 3.** (ing). Results of the Linear Mixed-Effects Model Predicting Professionalism Rating by (ing) guise for the British Speaker in the North of England (*N* = 44)

Factors	Estimate	Std. Error	*df*	*t* Value	Pr(>|t|)
(Intercept)	1.96	0.12	65	16.80	<0.001***
guise	0.001	0.001	263	0.53	0.59

Asterisks in statistical testing indicate significance levels: *p < 0.05, **p < 0.01, ***p < 0.001.

In Pennsylvania, in turn, guise did have a significant main effect on listener evaluations, regardless of which speaker participants heard. A relatively higher number of [ɪn]-tokens resulted in significantly more negative ratings of the British (*Est.* = 0.007, *p* < .001, see Table 4) as well as the American speaker (*Est.* = 0.005, *p* < .001, see Table 5). A positive value indicates that an increase in [ɪn]-tokens results in more negative professionalism ratings. As indicated by non-significantly lower AIC values (BritPenn: linear: AIC = 920, *r*^2^ = 71.00, logarithmic: AIC = 925, *r*^2^ = 71.00 and AmPenn: linear: AIC = 1018, *r*^2^ = 64.00, logarithmic: AIC = 1020, *r*^2^ = 64.00), the response patterns are best described as linear, rather than logarithmic, for both SpeakerRegion-groups in the US context (see Figure 1, upper graphs (a), dark blue lines with circles).

**Table 4.** (ing). Results of the Linear Mixed-Effects Model Predicting professionalism rating by (ing) guise for the British Speaker in Pennsylvania (*N* = 49)

Factors	Estimate	Std. Error	*df*	*t* Value	*p*-value
(Intercept)	2.42	0.24	56	9.97	<0.001***
guise	0.007	0.002	292	3.90	<0.001***
gender = woman	−0.30	0.35	56	−0.85	0.40
guise : gender = woman	−0.006	0.002	292	−2.46	0.01*

Asterisks in statistical testing indicate significance levels: *p < 0.05, **p < 0.01, ***p < 0.001.

**Table 5.** (ing). Results of the Linear Mixed-Effects Model Predicting professionalism rating by (ing) guise for the American Speaker in Pennsylvania (*N* = 45)

Factors	Estimate	Std. Error	*df*	*t* Value	*p*-value
(Intercept)	3.03	0.22	55	13.83	<0.001***
guise	0.005	0.002	269	2.70	0.007**

Asterisks in statistical testing indicate significance levels: *p < 0.05, **p < 0.01, ***p < 0.001.

In sum, these results show a regional difference between England (no significant effect of guise) and Pennsylvania (significant effect of guise on professionalism ratings), which provides further substantiation of previous findings by Labov et al. (2011) for the US context and Levon and Fox (2014) for the UK context. In contrast to Labov et al. (2011), however, a logarithmic distribution did not prove a significantly better description of the response pattern observed in the data.

Considering that our main focus is the effect of guise or interactions with it, we also tested for significant interaction terms in each of the four SpeakerRegion-specific models. We found that the British speaker in the South of England (see Table 2, *Est.* = −0.004, *p* < .03) is rated significantly better, as [ɪn]-tokens increase, by participants who show a high sensitivity to the expressive behavior of others (as indicated by a high score on the sebo-subscale of the Self-Monitoring Scale (SMS). Also, the British speaker is rated significantly better by women in Pennsylvania (*Est.* = −0.006, *p* < .01, see Table 4). Predictors which were *not* found to be significant but improved the overall model fit were included in the final model, for example, listeners’ pragmatic language ability (pragmatic_language: *Est.* = −0.002, *p* = .10).

The upper graphs (a) in Figure 1 not only include the distribution pattern for those respondents who did not become aware of the attitude target (blue lines), they also plot the response pattern for those participants who did identify the attitude target correctly (light pink lines with triangles). For all four SpeakerRegion-groups, distributions differ visibly between the ‘aware’ (light pink with triangles) and ‘unaware’ (dark blue with circles) listener groups. Unfortunately, responses are technically too few to include an interaction term between guise and awareness in a meaningful way in individual models. Nonetheless, we experimented with a second set of models and, this time, included all participants (see Tables A5-A8). An interaction term between guise and awareness was meant to reveal if the significant main effect for guise remains a significant predictor and provide statistical evidence for the factor of awareness, which it did. awareness is significant in interaction with guise in three out of four models (it is not significant in the model with only one aware participant, see Table A7). This indicates that participants who became aware of the attitude target rate the increase in [ɪn]-tokens significantly more negatively than those who did not realize what the study way about.

### 4.2. (t)-Deletion

Similar to the experiments on (ing), results for (t)-deletion are based on four related experiments: using the same test passage and research design, we collected responses of fifty participants from the North of England, fifty participants from the South of England, and twenty-five participants from Pennsylvania, who all heard the British speaker. We also collected responses from another group of twenty-five participants from Pennsylvania, who heard and rated the American speaker. The number of participants was smaller for experiments in the US context since initial experiments did not indicate any significant effect of frequency differences of deleted-[t] on listener evaluation, which made it unlikely that further experiments with more participants would change this picture.

**Figure 2.**
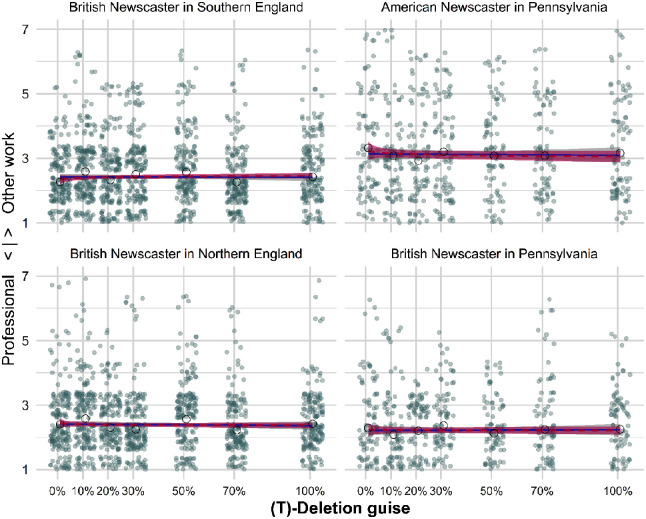
professionalism rating by (t)-Deletion guise for Each SpeakerRegion Constellation. Linear Model Fits Are Shown With the Unbroken Lines and Logarithmic Fits With Dashed Lines

We found no significant main effect for guise in our models (see Appendix, section “Further Results of Mixed-Effects Models,” Tables A1-A4). We observe a flat response pattern in all regions (see Figure 2). None of the respondents became aware of the attitude target. Thus, results for (ing) and (t)-deletion are markedly different.

## 5. Discussion

These results now allow us to make some generalizations and answer our research questions regarding the evaluation of quantitative differences in the use of [ɪn] and deleted-[t] in England and Pennsylvania. In particular, we explored (1) whether there is a significant effect of token-frequency in these regions, (2) what the nature of the response pattern is, and (3) how awareness of the attitude target affects evaluation.

As for (ing), our results indicate that [ɪn]-frequency affects listener evaluation in Pennsylvania significantly, regardless of which speaker was heard. In England, however, this effect was not attestable. These results thus substantiate findings from previous studies, for example, Labov et al. (2011), who found [ɪn]-frequency to be a significant predictor in the US, and Levon and Fox (2014), who did not find a significant effect of [ɪn]-frequency on professionalism ratings in the British context. While there are clear parallels between our results and previous findings, there are also a number of important differences, which offer fresh perspectives on the study of sociolinguistic monitoring in different locales.

First, conclusions in previous research about the social meanings of variation in (ing) in the US context as opposed to the UK are based on studies with similar research designs, but with slight divergences in their implementation. Levon and Fox (2014:196) used a test passage for their monitoring experiments that resembled the structure of Labov et al.’s (2011) original text but differed from it in length (thirty-six words longer) and with regard to the topics of the headlines (adapted to the British context); naturally, they also used a different speaker. Our study, in turn, is the first to offer a direct comparison of listener evaluations regarding variation in (ing) as we used the same set of guises across all three locales. In that sense, it offers robust conclusions about cross-regional patterns of listener evaluations and the social meanings of (ing). It attests clearly to British listeners’ insensitivity toward variation in (ing), whereas US listeners seem to track and evaluate varying [ɪn]-frequencies differently, even when produced by a British speaker.

Second, Labov et al. (2011) are often cited for having found a logarithmic response pattern in listener evaluation. While a logarithmic model did indeed prove to be the best fit for 38 percent of their data (all listeners, regardless of age group), a substantial share of their data (31 percent) were best described by a linear model and another 31 percent showed neither a linear nor a logarithmic pattern (see quantitative overview by Levon & Fox 2014:192; see also Labov et al. 2011:453). Their results are, thus, less straightforward than originally assumed, which makes finding a linear distribution quite plausible. In fact, our results indicate that neither the logarithmic nor the linear model are a significantly better fit for our data; they are very similar.

Third, the regression lines for the US participants (excluding those who became aware) only show a slightly increased slope and do not span across several evaluation points as it did in Labov et al. (2011) where it ranged from below two to above five. However, once we consider the response patterns of those participants who became aware of the attitude target (see Figure 1, upper graphs (a), pink lines with triangles), the response patterns are much more in line with those found by Labov et al. (2011).

Fourth, our study suggests that the question of whether those participants who became aware of the attitude target respond differently to varying [ɪn]-frequencies is crucial. Excluding the British speaker in Pennsylvania, where only one person became aware of the attitude target, all other models show a significant interaction term between guise and awareness, indicating that those participants who realized what the study was about rated the guises significantly differently as [ɪn]-frequencies increased (see Tables A5–A8 in the Appendix, section “Further Results of Mixed-Effects Models”). This is, perhaps, not surprising as it allows those participants to react knowingly to frequency differences and select a wider spread of ratings. As emphasized in section 4.1, the number of participants who did become aware is very low, which makes it difficult to include awareness in the statistical analysis in a meaningful way. If the four models for the unaware respondents (Tables 2–5) are compared with an equivalent set of models where all participants are included—without an interaction term for guise and awareness—results look decidedly different, indicating a significant or near-significant effect for Guise for all locales (see Tables A9–A12, in the Appendix, section “Further Results of Mixed-Effects Models,” see also Figure 1, lower graphs (b)). Thus, it becomes apparent that assessing listeners’ awareness and accounting for it in the statistical analysis is an indispensable move to make in future attitudes research.

Our conclusion raises an important question: despite the parallels in results, why do we observe differences in our data from the findings of Labov et al. (2011) regarding the range of professionalism-ratings and distribution in the US? We believe there are two reasons for this. In contrast to Labov et al. (2011), whose respondents were primarily students, our participants are of varying ages and backgrounds and are predominantly not students. Thus, our participant base differs from previous studies. Furthermore, Labov et al. also found evidence of a linear distribution pattern for parts of their data (see Labov et al. 2011:453; Levon & Fox 2014:192).

The logarithmic distribution documented in Labov et al. (2011) is not a side effect of the method used, that is, listening to multiple versions of a similar test passage. Instead, the logarithmic distribution they found may be idiosyncratic to their data: the methodological procedure may have differed in unpublished details from ours, which may have resulted in more participants in the original study to have become aware of the attitude target. This would explain the more prominent logarithmic distribution found by Labov et al. and that of participants who have become aware of the attitude target in our study.

Let us next consider results for (t)-deletion. While our findings for (ing) generally align with what we would expect based on production research and some previous perception studies, results for (t)-deletion were not in line with our expectations. As (t)-deletion was found to be subject to style-shifting (e.g., Baranowski & Turton 2020:16-17), one would expect sociolinguistic monitoring to take place, allowing speakers and listeners to track the frequency of standard/non-standard features accordingly. At the same time, our review of variables suggested that the difference in social stratification and general comment is less pronounced for deleted-[t] than for use of [ɪn]. As a result, we would expect a smaller effect of variant frequency for (t)-deletion than for (ing), which is indeed the case—at least for the US data.

Our finding that the frequency of deleted-[t] had no significant effect on listener evaluation across locales can be explained by the observation that, acoustically, deleted-[t] is harder to notice than [ɪn]. This can be seen in identification tasks implemented in the present study and in a further related set of experiments that looked more holistically at the noticeability, identification, and discrimination of sociolinguistic variants in England (Schleef, Roth, Mackay & Pflaeging forthcoming). Results for the present groups of respondents show that they are generally able to identify variants of (ing) and (t)-deletion correctly in read-out sentences and when presented with respective answer options: 86.2 percent of all participants identified the (ing)-variants correctly; 83.2 percent of all participants identified the variants of (t)-deletion correctly. If results for the individual variants are considered, however, it becomes apparent that the identification of deleted-[t] proved the most difficult: only 68.3 percent of deleted-[t]-tokens were identified correctly, whereas 98 percent of [t]-tokens were heard correctly. In contrast, the results for the two variants of (ing) were very similar to one another ([ɪn] 88.2 percent correct, [ɪŋ] 84.2 percent correct). The fact that the response pattern for (t)-deletion is flat and that not a single participant became aware of deleted-[t] in the present study is in line with these results.

Still, this raises the question of how speakers manage style-shifting—for (t)-deletion as well as (ing) in Britain—when sociolinguistic monitoring is not sensitive to such stylistically variable features? For one, production and perception may not necessarily align in monitoring processes. In addition, Vaughn (2022a) has argued that the kinds of matched-guise experiments as used in most previous studies on sociolinguistic monitoring, including our own, are run in incongruent styles, that is, informal features are spliced into otherwise formal language. Vaughn and Kendall (2019) found that read-out [ɪn]-sentences differed from [ɪŋ]-sentences in several vowel and consonant realizations. Thus, informal features naturally occur together with other informal features. Vaughn (2022a) compared stylistically congruent and incongruent guises with respect to (ing) and found that guises where [ɪn] appears in a congruent style are rated as most accented. Most other studies in the sociolinguistic monitor paradigm have used guises that seem incongruent in style. In the current study, the effect of [ɪn] versus [ɪŋ] could be captured even in stylistically incongruent guises in the US context. However, given the results of our study, such a research design may not be sufficient for (ing) in Britain and (t)-deletion in England and Pennsylvania. In these locales, [ɪn] and pre-consonantal deleted-[t] appear to fall under the radar in incongruent guises when they occur without other informal features.

## 6. Conclusion

Campbell-Kibler (2016) argues that the Labovian cognitive model is much too simple to account for the insights of third-wave variationist sociolinguistics and calls for an urgent update. Nonetheless, dynamics observed in the tradition of the sociolinguistic monitor must be incorporated into this new model. In order to achieve a tight integration of findings on sociolinguistic monitoring, contradictions and open questions in this paradigm must first be addressed and explained. It is relatively clear that “socially informed monitoring” is occurring (Campbell-Kibler 2016:142), although the monitoring process may not necessarily be language-specific. Speakers do monitor their own speech in production. To what extent this applies to perception and in how far production- and perception-related monitoring inform each other remains an open question.

We believe we have achieved two goals in this study that further work on sociolinguistic monitoring should consider. First, this study has been able to address the question of cross-regional comparability, and we were able to substantiate previously observed patterns, using the same test passage and speaker in different locales. Results suggest that frequency-based professionalism-ratings of (ing) and (t)-deletion are similar in the UK, but are different in the US context. Of course, when considering other genres and other evaluative scales, results may differ. After all, evaluation and social meaning are multidimensional (Eckert 2008), and our analysis focused on perceived professionalism only. Thus, we cannot speak to whether the variables may index any other social meanings. Second, we have also collected some first evidence that the factor of awareness of the attitude target may affect the response rate; different awareness states seem to result in different evaluative distributions. While more data is needed for a more robust description of this pattern, our results stress the importance of eliciting whether participants in experiments have become aware of the attitude target and of incorporating this information into the analysis. Different awareness states may very well explain the different findings in studies conducted within the sociolinguistic monitoring paradigm and exploring them represents a crucial area of future investigation.

## Appendix

### Test Passages

#### (ing) Test Passage

*Good afternoon to you both. Yes, it’s the largest construction expo so far and I’ve been* ***(1) testin’*** *this exo-skeleton here. For some of the issues the construction industry is* ***(2) facin’*** *these days, this is one of the best proposals. Other issues are, of course: Are we* ***(3) makin’*** *things quick enough? Have we developed robust future-proof methods? And, of course, are they sustainable? The construction industry has been* ***(4) workin’*** *hard toward many solutions and this structure here is one of the strongest proposals so far. Companies have been* ***(5) referrin’*** *broadly to this method as pre-fab or pre-cast construction. Structures such as the one I’m* ***(6) standin’*** *by usually take more than five days to go up, whereas recent projects are* ***(7) bein’*** *finished much faster. Companies essentially manufacture each piece separately and then assist contractors with the build. This has convinced various types of stakeholders: The Builder’s Trust, for example, has been* ***(8) puttin’*** *some gentle pressure behind this innovative new method and its promotion. It’s* ***(9) offerin’*** *supplemental grant funds for projects which plan to use pre-fab construction methods. And representatives from most companies I’ve spoken to today are* ***(10) explorin’*** *this technology now. Will this be the Holy Grail for the industry? Let’s speak to John.*

#### (t)-Deletion Test Passage

*Good afternoon to you both. Yes, it’s the* ***(1) larges’*** *construction expo so far and I’ve been testing this exo-skeleton here. For some of the issues the construction industry is facing these days, this is one of the* ***(2) bes’*** *proposals. Other issues are, of course: Are we making things quick enough? Have we developed* ***(3) robus’*** *future-proof methods? And, of course, are they sustainable? The construction industry has been working hard toward many solutions and this structure here is one of the* ***(4) stronges’*** *proposals so far. Companies have been referring broadly to this method as pre-fab or* ***(5) pre-cas’*** *construction. Structures such as the one I’m standing by usually take more than five days to go up, whereas* ***(6) recen’*** *projects are being finished much faster. Companies essentially manufacture each piece separately and then* ***(7) assis’*** *contractors with the build. This has convinced various types of stakeholders: The Builder’s* ***(8) Trus’***, *for example, has been putting some gentle pressure behind this innovative new method and its promotion. It’s offering supplemental* ***(9) gran’*** *funds for projects which plan to use pre-fab construction methods. And representatives from* ***(10) mos’*** *companies I’ve spoken to today are exploring this technology now. Will this be the Holy Grail for the industry? Let’s speak to John.*

### Questionnaire

**1** INTRODUCTION
  - **MOOD:** *How are you feeling today?*
    ○ [rating scale] −5 | −4 | −3 | −2 | −1 | 0 | +1 | +2 | +3 | +4 | +5
  - **INSTRUCTIONS, AUTO-PLAY SETTINGS, TRIAL QUESTION**
**2** EVALUATION OF 7 VERSIONS OF AN ON-SITE NEWS REPORT
  - **INSTRUCTIONS:** *In this part of the survey, you are asked to listen to and evaluate seven versions of a trial news report recorded with an on-site reporter in training. One of the recordings is to be submitted with a job application as an on-site reporter in England. Note that you’ll be hearing the same speaker producing the same report several times. Each recording is available on a separate page and will play automatically. You can listen to each recording only once. Listen to the full recording first, then rate it on a scale. The Next-button will appear once the recording has played through. Please respond to the tasks fully and honestly. If you are unsure, choose the idea that comes to your mind first.*
  - **TRIAL QUESTION**
  - **7 VERSIONS OF THE ON-SITE REPORT**
    ○ [rating scale] 1 (try some other line of work) | 2 | 3 | 4 | 5 | 6 | 7 (perfectly professional)
**3** AWARENESS QUESTIONS
  - **AWARENESS1:** *If we told you this study was about a specific language feature, which one would you think it was?*
    ○ [short free text]
  - **AWARENESS2:** *When did you realize that this study was about a specific language feature?*
    ○ *after about 25% of the recordings*
    ○ *after about 50% of the recordings*
    ○ *after about 75% of the recordings*
    ○ *after about 100% of the recordings*
    ○ *I didn’t realize it was about a specific language feature.*
**4** IDENTIFICATION TASKS
  - **TRIAL QUESTION**
  - ID1: *You will hear a recording of the word reading* (or *agent*, respectively).
    *How was it pronounced?*
      ○ *reading* (or *agent*)
      ○ *readin’* (or *agen’*)
      ○ *I don’t know.*
  - **ID2:** *You will hear a recording of the word running* (or *client*, respectively).
    *How was it pronounced?*
      ○ *running* (or *client*)
      ○ *runnin’* (or *clien’*)
      ○ *I don’t know.*
  - **ID3:** *You will hear a recording of the word talking* (or *comment*, respectively). *How was it pronounced?*
    ○ *talking* (or *comment*)
    ○ *talkin’* (or *commen’*)
    ○ *I don’t know.*
  - **ID4:** *You will hear a recording of the word walking* (or *moment*, respectively). *How was it pronounced?*
    ○ *walking* (or *moment*)
    ○ *walkin’* (or *momen’*)
    ○ *I don’t know.*
**5** PSYCHOMETRIC TESTS
  - **SMS** [13 items + 2 attention checks]
    ○ 1 (strongly disagree) | 2 | 3 | 4 | 5 (strongly agree)
  - **BAPQ** [36 items + 2 attention checks]
    ○ 1 (very rarely) | 2 (rarely) | 3 (occasionally) | 4 (somewhat often) | 5 (often) | 6 (very often)
  - **MCPR** [10 items + 2 attention checks]
    ○ 1 (strongly disagree) | 2 | 3 | 4 | 5 | 6 | 7 (strongly agree)
**6** USAGE
  - **USAGE:** *In some of the recordings, the speaker pronounced reading as readin’. Do you ever use this type of pronunciation?*
    ○ *Yes, regularly.*
    ○ *Yes, sometimes.*
    ○ *No, never.*
**7** FATIGUE
  - **FATIGUE:** *How interesting/boring did you find this study?*
    - ○ 1 (very interesting) | 2 | 3 | 4 | 5 | 6 | 7 (very boring)
**8** DEMOGRAPHICS
  • **GENDER:** *Which of the following best describes your gender identity?*
    ○ *woman*
    ○ *man*
    ○ *non-binary*
    ○ *prefer not to say*
    ○ *other*: [short free text]
  • **AGE:** *How old are you?*
    ○ [short numerical input]
  • **CLASS:** *What social class do you think you belong to?*
    ○ *lower working-class*
    ○ *upper working-class*
    ○ *lower middle-class*
    ○ *upper middle-class*
    ○ *lower upper-class*
    ○ *upper upper-class*
  • **LANGUAGE:** *Is English your native/first language*
    ○ yes
    ○ *no* . . . if ‘no’ . . .
   ■ *What language would you consider your native/first language?*
    • [short free text]
   ■ *How many years have you lived in the UK/US?*
    • [short numerical input]
  • [only UK] **REGION:** *What is your current place of residence?*
    ○ *North East, England (Tees Valley, Durham, Northumberland and Tyne and Wear)*
    ○ *North West, England (Cumbria, Greater Manchester, Lancashire, Merseyside)*
    ○ *Yorkshire and the Humber, England (East Riding, North Lincolnshire and Yorkshire)*
    ○ *London, England*
    ○ *South East, England (Berkshire, Buckinghamshire, and Oxfordshire, Surrey, Sussex, Kent, Hampshire and Isle of Wight)*
    ○ *South West, England (Gloucestershire, Wiltshire and Bristol/Bath area, Dorset and Somerset, Cornwall and Isles of Scilly, Devon)*
  • **POST-/ZIP-CODE:**
    *Please provide the first three characters of your postcode/US zip code.*
      ○ [short free text]
  • **AUDIO:** *What type of audio output device did you use for this study?*
    ○ *headphones*
    ○ *in-built computer speakers*
    ○ *external speakers*

### Further Results of Mixed-Effects Models

**Table A1.** (t)-deletion. Results of the Linear Mixed-Effects Model Predicting professionalism rating by (t)-Deletion guise for the British Speaker in the South of England (*N* = 50)

Factors	Estimate	Std. Error	*df*	*t* Value	*p*-value
(Intercept)	2.42	0.14	51	17.59	<0.001***
guise	−0.001	<0.001	1349	−0.16	0.875

Asterisks in statistical testing indicate significance levels: **p* < 0.05, ***p* < 0.01, ****p* < 0.001.

**Table A2.** (t)-deletion. Results of the Linear Mixed-Effects Model Predicting professionalism rating by (t)-Deletion guise for the British Speaker in the North of England (*N* = 50)

Factors	Estimate	Std. Error	*df*	*t* Value	*p*-value
(Intercept)	2.41	0.14	50	16.70	<0.001***
guise	−0.001	<0.001	1349	−1.18	0.24

Asterisks in statistical testing indicate significance levels: **p* < 0.05, ***p* < 0.01, ****p* < 0.001.

**Table A3.** (t)-deletion. Results of the Linear Mixed-Effects Model Predicting professionalism rating by (t)-Deletion guise for the British Speaker in Pennsylvania (*N* = 25)

Factors	Estimate	Std. Error	*df*	*t* Value	*p*-value
(Intercept)	2.21	0.19	25	11.65	<0.001***
guise	<0.001	<0.001	674	0.26	0.799

Asterisks in statistical testing indicate significance levels: **p* < 0.05, ***p* < 0.01, ****p* < 0.001.

**Table A4.** (t)-deletion. Results of the Linear Mixed-Effects Model Predicting professionalism rating by (t)-Deletion guise for the American Speaker in Pennsylvania (*N* = 25)

Factors	Estimate	Std. Error	*df*	*t* Value	*p*-value
(Intercept)	3.38	1.54	22	2.20	0.039*
guise	−0.0004	<0.001	674	−0.38	0.703
pragmatic_language	−0.02	<0.01	22	−2.61	0.016*
rigid	0.01	<0.01	22	1.64	0.114

Asterisks in statistical testing indicate significance levels: **p* < 0.05, ***p* < 0.01, ****p* < 0.001.

**Table A5.** (ing). Results of the Linear Mixed-Effects Model Predicting professionalism rating by (ing) guise for the British Speaker in the South of England (*N* = 50), Including an Interaction Term Between Guise and Awareness

Factors	Estimate	Std. Error	*df*	*t* Value	*p*-value
(Intercept)	2.41	0.24	49	10.18	<0.001***
guise	0.001	0.001	298	0.64	0.53
aware = yes	0.22	0.70	53	0.32	0.75
age group = younger	0.12	0.32	45	0.36	0.72
age group = older	2.71	1.13	45	2.41	0.02*
pragmatic_language	−0.25	0.16	45	−1.58	0.12
guise : aware = yes	0.02	0.005	298	5.28	<0.001***

Asterisks in statistical testing indicate significance levels: **p* < 0.05, ***p* < 0.01, ****p* < 0.001.

**Table A6.** (ing). Results of the Linear Mixed-Effects Model Predicting professionalism rating by (ing) guise for the British Speaker in the North of England (*N* = 50), Including an Interaction Term Between Guise and Awareness

Factors	Estimate	Std. Error	*df*	*t* Value	*p*-value
(Intercept)	1.95	0.12	66	16.06	<0.001***
guise	0.001	0.001	296	0.82	0.41
aware = yes	0.05	0.37	66	0.13	0.90
motivation_overall	0.17	0.13	67	1.27	0.21
amsp	−0.20	0.13	67	−1.56	0.12
guise : aware = yes	0.02	0.004	296	5.88	<0.001***
guise : motivation_overall	−0.003	0.001	296	−2.33	0.02*
guise : amsp	0.003	0.001	296	2.32	0.02*

Asterisks in statistical testing indicate significance levels: **p* < 0.05, ***p* < 0.01, ****p* < 0.001.

**Table A7.** (ing). Results of the Linear Mixed-Effects Model Predicting professionalism rating by (ing) guise for the British Speaker in Pennsylvania (*N* = 50), Including All Participants

Factors	Estimate	Std. Error	*df*	*t* Value	*p*-value
(Intercept)	2.05	0.28	46	7.44	<0.001***
guise	0.01	0.001	1348	8.22	<0.001***
pragmatic_language	−0.40	0.16	45	−2.52	0.02*
age group = younger	0.90	0.32	45	2.84	0.02**
age group = older	0.35	0.66	45	0.54	0.6
gender = woman	−0.31	0.32	47	−0.95	0.35
guise : gender = woman	−0.01	0.001	1348	−4.80	<0.001***

Asterisks in statistical testing indicate significance levels: **p* < 0.05, ***p* < 0.01, ****p* < 0.001.

**Table A8.** (ing). Results of the Linear Mixed-Effects Model Predicting Rating on the Professionalism-Scale by (ing) guise for the American Speaker in Pennsylvania (*N* = 50), Including an Interaction Term Between Guise and Awareness

Factors	Estimate	Std. Error	*df*	*t* Value	*p*-value
(Intercept)	3.03	0.22	59	14.10	<0.001***
guise	0.005	0.002	298	2.76	0.006**
aware = yes	−1.31	0.68	59	−1.92	0.06
guise : aware = yes	0.02	0.01	298	2.85	0.005**

Asterisks in statistical testing indicate significance levels: **p* < 0.05, ***p* < 0.01, ****p* < 0.001.

**Table A9.** (ing). Results of the Linear Mixed-Effects Model Predicting Rating on the Professionalism-Scale by (ing) guise for the British Speaker in the South of England (*N* = 50), Without Interaction Terms*

Factors	Estimate	Std. Error	*df*	*t* Value	*p*-value
(Intercept)	3.38	0.68	45	4.98	<0.001***
guise	0.002	0.001	299	1.91	0.06
aware = yes	1.23	0.67	45	1.85	0.07
pragmatic_language	−0.004	0.003	45	−1.58	0.12
age group = younger	0.12	0.32	45	0.36	0.72
age group = older	2.71	1.13	45	2.41	0.02*

* In line with all other models presented in this paper, we are giving the results of the linear model here where guise was merely approaching significance. The equivalent logarithmic model, however, indicated guise as a significant predictor (*p* = .047).

Asterisks in statistical testing indicate significance levels: **p* < 0.05, ***p* < 0.01, ****p* < 0.001.

**Table A10.** (ing). Results of the Linear Mixed-Effects Model Predicting Rating on the Professionalism-Scale by (ing) guise for the British Speaker in the North of England (*N* = 50), Without Interaction Terms

Factors	Estimate	Std. Error	*df*	*t* Value	*p*-value
(Intercept)	1.84	0.11	53	16.68	<0.001***
guise	0.00	0.0006	1349	6.21	<0.001***
aware = yes	0.89	0.31	48	2.86	0.006**

Asterisks in statistical testing indicate significance levels: **p* < 0.05, ***p* < 0.01, ****p* < 0.001.

**Table A11.** (ing). Results of the Linear Mixed-Effects Model Predicting Rating on the Professionalism-Scale by (ing) guise for the British Speaker in Pennsylvania (*N* = 50), Without Interaction Terms

Factors	Estimate	Std. Error	*df*	*t* Value	*p*-value
(Intercept)	2.71	0.44	46	6.16	<0.001***
guise	0.004	0.0006	1349	6.76	<0.001***
pragmatic_language	−0.003	0.002	46	−2.08	0.043*
age group = younger	0.99	0.32	46	3.12	0.003**
age group = older	0.23	0.67	46	0.35	0.729

Asterisks in statistical testing indicate significance levels: **p* < 0.05, ***p* < 0.01, ****p* < 0.001.

**Table A12.** (ing). Results of the Linear Mixed-Effects Model Predicting Rating on the Professionalism-Scale by (ing) guise for the American Speaker in Pennsylvania (*N* = 50), Without Interaction Terms

Factors	Estimate	Std. Error	*df*	*t* Value	*p*-value
(Intercept)	2.90	0.20	51	14.76	<0.001***
guise	0.006	0.0008	1349	8.09	<0.001***

Asterisks in statistical testing indicate significance levels: **p* < 0.05, ***p* < 0.01, ****p* < 0.001.

## Notes

1. To ensure comparability with previous research (e.g., Levon & Fox 2014), we clarified the exact order of token splicing in a personal correspondence with Erez Levon (April 2022). Following Levon and Fox’s procedure, we first replaced token 5 (10 percent guise), then also 7 (20 percent guise), then 3 (30 percent guise), then 9 and 1 (50 percent guise), then tokens 6 and 4 (70 percent guise), and finally tokens 2, 8, and 10 (100 percent guise).

2. Test sentences were recorded with a female speaker in her mid-forties. Originally from Liverpool in England, her accent is clearly Northern but may not allow listeners to associate her with a specific city.

3. *Prolific* does not support filtering for a particular city within Pennsylvania State, for example, Philadelphia, and participants’ zip code data suggests that listeners were from different parts of the state. We thus acknowledge that social evaluation may have been influenced by differences in listeners’ linguistic background (e.g., dialect differences between Southeastern versus Southwestern Pennsylvania), as pointed out by a reviewer.

4. We decided to process the age data as a categorical factor as models with age group proved to be a better fit for our data than models that included age as a continuous variable.

5. As zero cannot be log-transformed, a constant number was added to each guise-value (+1).
